# Influence of Burning-Induced Electrical Signals on Photosynthesis in Pea Can Be Modified by Soil Water Shortage

**DOI:** 10.3390/plants11040534

**Published:** 2022-02-17

**Authors:** Lyubov Yudina, Ekaterina Gromova, Marina Grinberg, Alyona Popova, Ekaterina Sukhova, Vladimir Sukhov

**Affiliations:** Department of Biophysics, N.I. Lobachevsky State University of Nizhny Novgorod, 603950 Nizhny Novgorod, Russia; lyubovsurova@mail.ru (L.Y.); kater333@inbox.ru (E.G.); mag1355@yandex.ru (M.G.); silverkumiho@mail.ru (A.P.); n.catherine@inbox.ru (E.S.)

**Keywords:** electrical signals, local burning, soil drought, water shortage, photosynthetic CO_2_ assimilation, non-photochemical quenching, linear electron flow, cyclic electron flow around photosystem I, leaf stomatal conductance

## Abstract

Local damage to plants can induce fast systemic physiological changes through generation and propagation of electrical signals. It is known that electrical signals influence numerous physiological processes including photosynthesis; an increased plant tolerance to actions of stressors is a result of these changes. It is probable that parameters of electrical signals and fast physiological changes induced by these signals can be modified by the long-term actions of stressors; however, this question has been little investigated. Our work was devoted to the investigation of the parameters of burning-induced electrical signals and their influence on photosynthesis under soil water shortage in pea seedlings. We showed that soil water shortage decreased the amplitudes of the burning-induced depolarization signals (variation potential) and the magnitudes of photosynthetic inactivation (decreasing photosynthetic CO_2_ assimilation and linear electron flow and increasing non-photochemical quenching of the chlorophyll fluorescence and cyclic electron flow around photosystem I) caused by these signals. Moreover, burning-induced hyperpolarization signals (maybe, system potentials) and increased photosynthetic CO_2_ assimilation could be observed under strong water shortage. It was shown that the electrical signal-induced increase of the leaf stomatal conductance was a potential mechanism for the burning-induced activation of photosynthetic CO_2_ assimilation under strong water shortage; this mechanism was not crucial for photosynthetic response under control conditions or weak water shortage. Thus, our results show that soil water shortage can strongly modify damage-induced electrical signals and fast physiological responses induced by these signals.

## 1. Introduction

Long-distance electrical signals (ESs), which are induced by local actions of stressors and propagate into non-irritated zones, are an important mechanism of induction of systemic adaptation response in plants [1,2,3,4,5,6,7,8]. Three types of electrical signals including variation potential (VP), action potential (AP), and system potential (SP) are often considered to be present in higher plants [6,7,8]. VP is a long-term “depolarization signal” (minutes and tens of minutes) [9,10] which is induced by local damage and has irregular shape (long-term depolarization, fast initial depolarization, and “AP-like” spikes can be observed); its parameters are dependent on the distance from the damaged zone. A transient inactivation of H^+^-ATPase in the plasma membrane is considered to be the main mechanism of VP generation [9,10]. AP is a short-term depolarization signal (mainly, seconds and tens of seconds) [4,11,12,13] which is induced by stimuli with weak and moderate intensity and has a spike shape; its parameters are not dependent on the distance from the irritated zone. The generation of AP is mainly related to transient activation of calcium, anion, and potassium channels [12,14,15]; however, a short-term inactivation of H^+^-ATPase can also participate in this electrical response [16]. SP is a long-term hyperpolarization signal (mainly, minutes and tens of minutes) [7,17,18] which often accompanies VP and is caused by transient activation H^+^-ATPase.

ESs can strongly influence physiological processes in plants [1,2,7,8]. It is probable that AP and VP induce similar physiological changes [7,8]: the stimulation of expression of defense genes [19,20,21,22,23], activation of production of stress phytohormones including abscisic acid, jasmonic acid, salicylic acid, and ethylene [23,24,25,26,27,28,29], modification of stomata opening, transpiration, and water content [30,31,32,33,34], activation of respiration [35,36,37], suppression of phloem loading [38,39] and phloem mass-flow [40,41,42], increasing ATP content [43], and many others. Photosynthesis is an important target of influence of AP and VP [44]. It is known that ESs decrease the CO_2_ flux into mesophyll cells [45] and suppress photosynthetic dark reactions [46,47], increase the non-photochemical quenching of the chlorophyll fluorescence (NPQ) [46,47,48,49,50], decrease the photosynthetic linear electron flow (LEF), and stimulate the cyclic electron flow around photosystem I (CEF) [51]. Inactivation of H^+^-ATPase [52,53] and changes in intra- and extracellular pH [54,55,56], which are related to VP and AP generation, are the probable mechanism of the induction of these photosynthetic changes. Increase of the plant tolerance to actions of stressors is an important result of ES-induced physiological changes [57,58,59,60,61,62,63,64]; ES-induced photosynthetic changes are probable to participate in this increase of plant tolerance [56,62,65,66]. 

Influence of SP on physiological processes (particularly, photosynthesis) has been little investigated. There are few works (e.g., [32,67]) which show that ESs with different directions (depolarization or hyperpolarization signals induced by different stimuli) induce changes in photosynthetic CO_2_ assimilation (A_CO2_) with different directions; alternatively, photosynthetic responses can be absent at a specific direction of the electrical signal [68]. These results show that specific photosynthetic responses, which differ from AP- and VP-caused responses, can be induced by SP. In contrast, other works [69] show that ESs with different directions induce similar suppression of A_CO2_; the last result is in a good accordance with our previous theoretical conclusion [7] based on apoplastic alkalization during SP [17]. Relations between the generation and propagation of SP and changes in plant tolerance to stressors have also been weakly investigated; earlier, we speculated that SP can positively influence plant tolerance [7] but the hypothesis requires further investigations. 

Thus, ESs (especially, VP and AP) are the important mechanism of fast plant responses on actions of stressors. This result can be used for development of new methods of revealing actions of stressors on plants based on both direct measurements of their electrical activity [70,71,72,73,74,75,76,77,78,79] and measurements plant reflectance which is strongly related to the physiological responses induced by ESs [34,80,81,82]. It can be expected that long-term changes in environmental conditions can modify the parameters of propagation of electrical signals and their influence on physiological processes. There are some works showing modification of ESs and the physiological responses under actions of specific environmental factors (e.g., [83] shows that ESs in plants are modified under action of ionizing radiation); however, the influence of many other long-term factors on the parameters of ESs and physiological responses has been weakly investigated. 

In the current work, we investigated the parameters of burning-induced ESs and photosynthetic responses, which were caused by these electrical signals, in pea seedlings under a soil water shortage because the water shortage can strongly influence photosynthesis and productivity in plants [84,85,86,87]. Burning-induced ESs were analyzed because these signals and their influence on photosynthesis in pea seedling under control conditions (well irrigated plants or plants cultivated in hydroponic medium) had been investigated in detail in our earlier works [50,51,52,55]. 

## 2. Results

### 2.1. Influence of Soil Water Shortage on Photosynthetic Parameters and Leaf Stomatal Conductance without Induction of Electrical Signals

**Figure 1:**
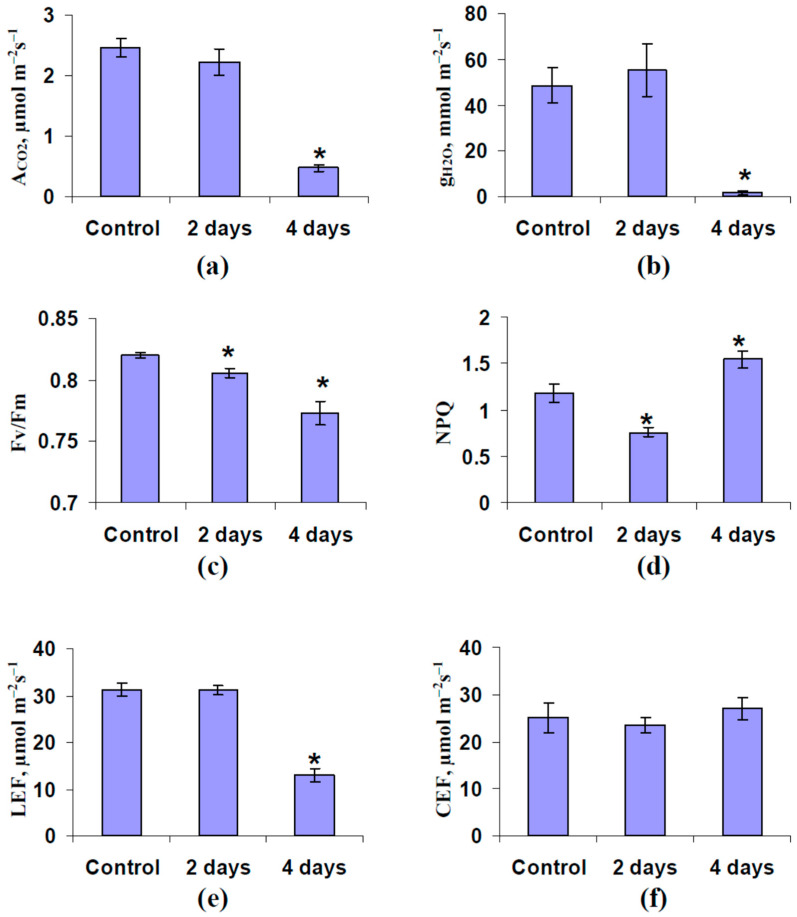
Influence of soil water shortage on the photosynthetic CO_2_ assimilation (A_CO2_) (**a**), leaf stomatal conductance (g_H2O_) (**b**), maximal quantum yield of photosystem II (Fv/Fm) (**c**), non-photochemical quenching of the chlorophyll fluorescence (NPQ) (**d**), photosynthetic linear electron flow (LEF) (**e**), and cyclic electron flow around photosystem I (CEF) (**f**) in pea seedlings (*n* = 6). *n* was the quantity of investigated seedlings. Water shortage was initiated by the termination of irrigation of plants. Parameters were measured in the second mature leaf. A_CO2_ was calculated as the difference between CO_2_ assimilation (A) under light and dark conditions. *, the parameter significantly differed from the one in control pea seedlings (*p* < 0.05, Student’s *t*-test).

The influence of soil water shortage on photosynthetic parameters and leaf stomatal conductance without induction of ESs was analyzed at the first stage of investigation (Figure 1). Soil water shortage was induced by termination of irrigation; in accordance with our previous results [88,89], this termination induced fast water loss by using a sand substrate for cultivation of the plants and can be used as a model of soil drought. 

It was shown that most of the investigated parameters were not significantly changed after 2 days of water shortage (Figure 1) excluding the maximal quantum yield of photosystem II and non-photochemical quenching; these parameters were decreased. In contrast, the photosynthetic CO_2_ assimilation, leaf stomatal conductance, maximal quantum yield of photosystem II, and linear electron flow were suppressed after 4 days of water shortage and non-photochemical quenching was increased, i.e., there were typical stress changes in the photosynthetic processes in the plants [85,90,91,92,93,94,95]. It was interesting that CEF, which could be also stimulated by the actions of stressors [85,96,97,98,99], was not significantly influenced by soil water shortage. 

### 2.2. Influence of Soil Water Shortage on Parameters of Burning-Induced Electrical Signals in Pea Seedlings

**Figure 2:**
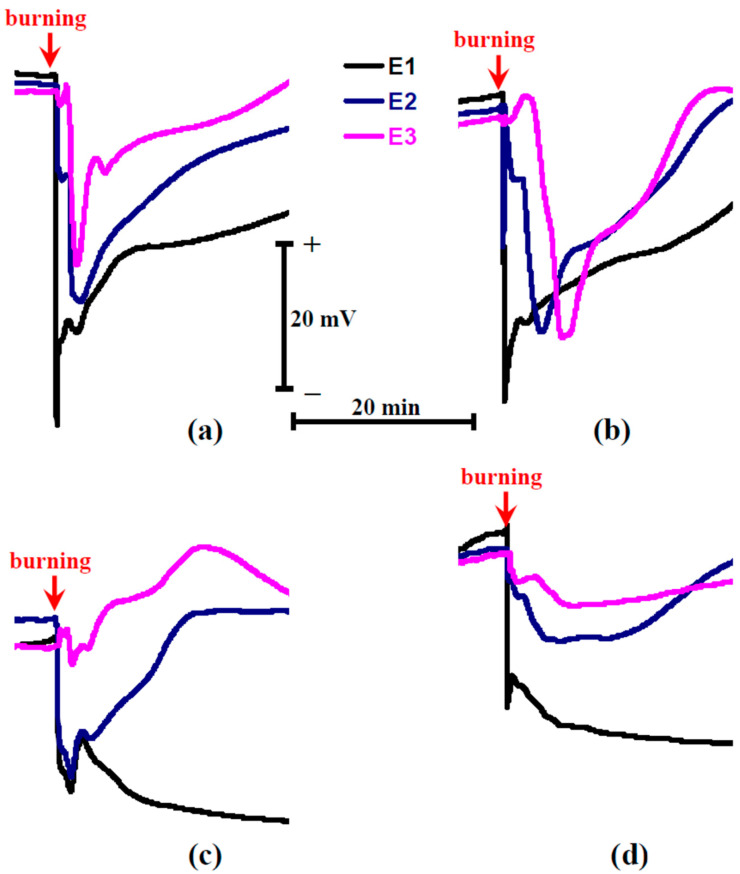
Records of burning-induced electrical signals in control pea seedlings (**a**), seedlings after 2 days of water shortage (**b**), and seedlings after 4 days of this shortage (**c**,**d**). Extracellular measurements in the stem near to the first mature leaf (E1), the stem near to the second mature leaf (E2), and leaflet of the second mature leaf (E3) are shown. Arrow marks the time of the burning of the first mature leaf. Figure 2c shows the hyperpolarization electrical signal in the second leaf which was observed in two peas from six plants after 4 days of water shortage; Figure 2d shows a weak depolarization electrical signal in the second leaf which was observed in four peas from six plants after 4 days of water shortage. Only depolarization signals were observed in control plants and plants after 2 days of water shortage.

The influence of the soil water shortage on the parameters of burning-induced ESs in pea seedlings was analyzed at the second stage of the investigation (Figure 2) by using extracellular measurements of electrical activity.

**Figure 3:**
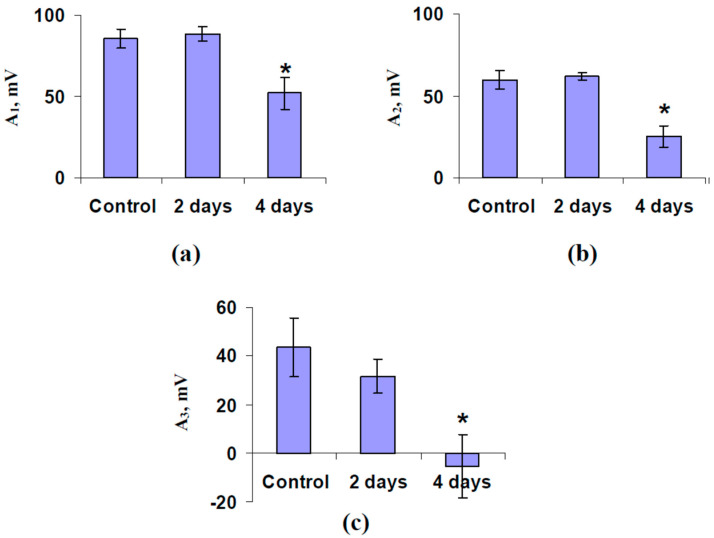
Influence of soil water shortage on the amplitudes of the burning-induced electrical signals in the stem near to the first mature leaf (A_1_) (**a**), the stem near to the second mature leaf (A_2_) (**b**), and leaflet of the second mature leaf (A_3_) (**c**) in pea seedlings (*n* = 6). The water shortage was initiated by the termination of irrigation of the plants. It was assumed that negative amplitudes corresponded to the hyperpolarization signal. *, the amplitude significantly differed from the one in the control pea seedlings (*p* < 0.05, Student’s *t*-test).

It was shown (Figure 2a) that the local burning induced typical VP (the depolarization signal) in pea seedlings under control conditions: duration was more than 20 min, shape was irregular, and amplitude was decreased on increasing the distance from the damage zone [7,10]. The burning-induced ESs, which were observed after 2 days of soil water shortage, were similar to the control depolarization signal. In contrast, ESs were modified after 4 days of water shortage; there were two types of signals: hyperpolarization signals (Figure 2c) and depolarization signals (Figure 2d) with decreased amplitude. The hyperpolarization signals were similar to SP [17]; e.g., strong hyperpolarization and large duration were observed. Analysis of the averaged amplitudes of the burning-induced ESs showed absence of a significant difference from amplitudes in control plants after 2 days of soil water shortage and a significant decrease of ES amplitude after 4 days of this shortage (Figure 3). 

Additionally, we analyzed the correlation coefficients (R) between the amplitudes of ESs in different parts of the plants, which were calculated on the basis of the electrical signals in all experimental seedlings. It was shown that R was 0.82 for the amplitudes in the stem near to the first leaf (A_1_) and ones in the stem near to the second leaf (A_2_), R was 0.52 for A_1_ and amplitudes in the second leaf (A_3_), and R was 0.50 for A_2_ and A_3_; all correlation coefficients were significant. This result showed that the parameters of ESs were very similar in the stem, but the electrical signals were changed after their propagation into leaves; it was in a good accordance with our previous results, which showed that the amplitudes of ESs were strongly decreased in leaves [43]. 

### 2.3. Influence of Soil Water Shortage on Parameters of Burning-Induced Changes in Photosynthetic Parameters and Leaf Stomatal Conductance

**Figure 4:**
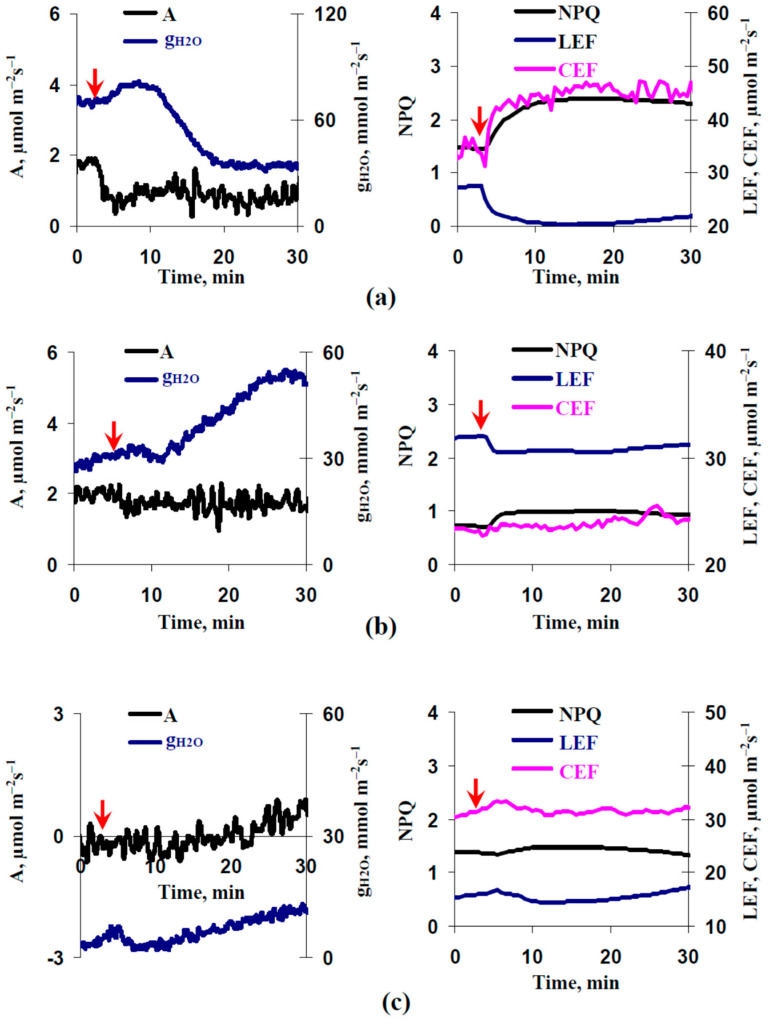
Records of burning-induced changes in CO_2_ assimilation (A), leaf stomatal conductance (g_H2O_), non-photochemical quenching (NPQ), photosynthetic linear electron flow (LEF), and cyclic electron flow around photosystem I (CEF) in control pea seedlings (**a**), seedlings after 2 days of soil water shortage (**b**), and seedlings after 4 days of this shortage (**c**) (*n* = 6). Parameters were measured in the second mature leaf. Arrow marks the time of the burning of the first mature leaf.

Figure 4 shows the records of the burning-induced changes in the photosynthetic parameters and leaf stomatal conductance in control pea seedlings and seedlings after 2 and 4 days of soil water shortage. It was shown that the local burning induced typical photosynthetic responses under control conditions (the decrease of CO_2_ assimilation and LEF and the increase of NPQ and CEF [51]). Magnitudes of changes in NPQ, LEF, and CEF were moderately decreased after 2 days of soil water shortage and were strongly decreased after 4 days of this shortage. Magnitude of suppression of the CO_2_ assimilation after 2 days of soil water shortage was similar to the control; in contrast, the local burning induced slow stimulation of the CO_2_ assimilation after 4 days of water shortage. It was additionally shown that the local burning induced large changes in g_H2O_; however, the directions of these changes were different in the various experimental plants. 

**Figure 5:**
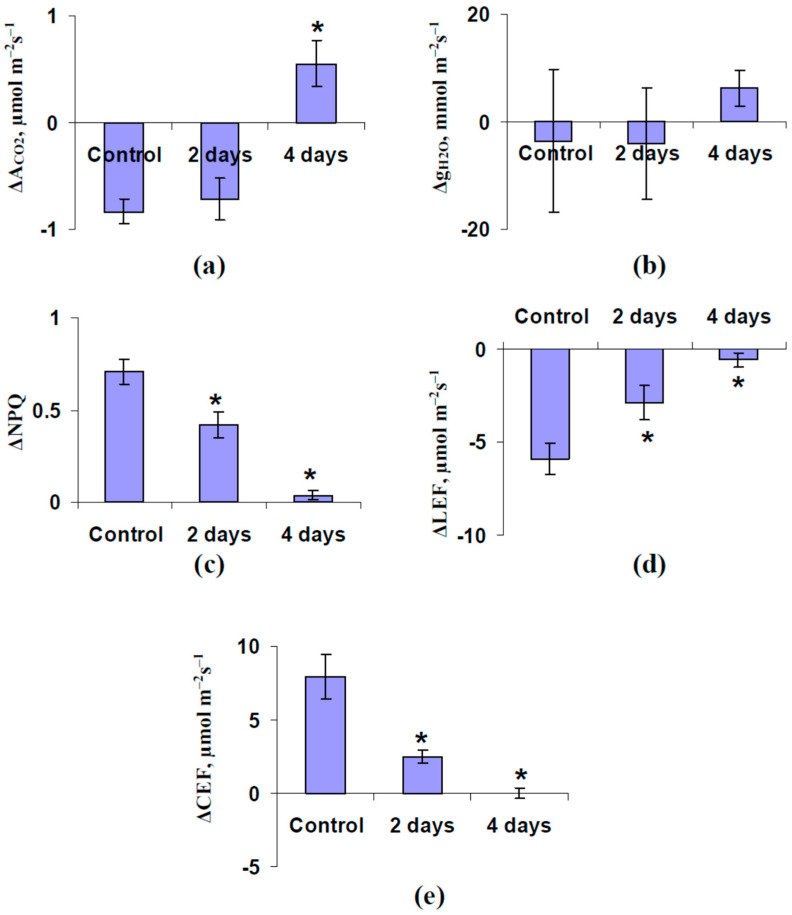
Influence of the soil water shortage on the burning-induced changes in photosynthetic CO_2_ assimilation (ΔA_CO2_) (**a**), leaf stomatal conductance (Δg_H2O_) (**b**), non-photochemical quenching (ΔNPQ) (**c**), photosynthetic linear electron flow (ΔLEF) (**d**), and cyclic electron flow around photosystem I (ΔCEF) (**e**) in pea seedlings (*n* = 6). The water shortage was initiated by the termination of irrigation of the plants. Parameters were measured in the second mature leaf; the first mature leaf underwent burning. *, the parameter significantly differed from the one in control pea seedlings (*p* < 0.05, Student’s *t*-test).

The analysis of the averaged magnitudes supported these results (Figure 5). It was shown that the local burning induced a significant decrease of A_CO2_ (ΔA_CO2_) under control conditions and after 2 days of soil water shortage, and a significant increase of this parameter after 4 days of this shortage (Figure 5a). The magnitudes of changes in NPQ (ΔNPQ) (Figure 5c), LEF (ΔLEF) (Figure 5d), and CEF (ΔCEF) (Figure 5e) were significantly decreased after 2 days (moderate decrease) and after 4 days (strong decrease) of soil water shortage. It was important that the averaged magnitude of changes in g_H2O_ (Δg_H2O_) had large error and did not differ from zero; the local burning induced an increase of g_H2O_ after only 4 days of soil water shortage (tendency).

**Figure 6:**
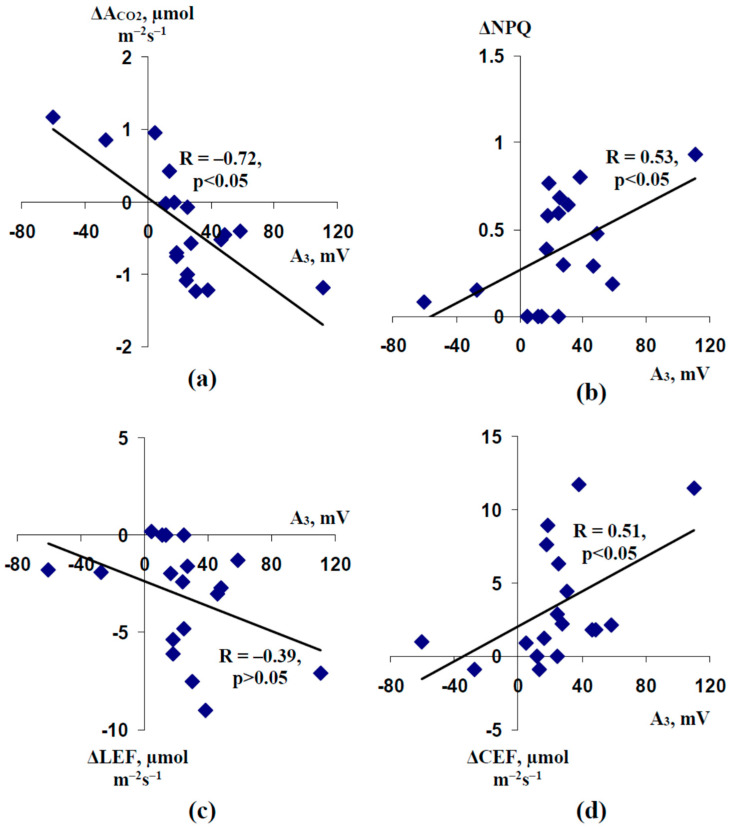
Dependences of burning-induced changes in photosynthetic CO_2_ assimilation (ΔA_CO2_) (**a**), non-photochemical quenching (ΔNPQ) (**b**), photosynthetic linear electron flow (ΔLEF) (**c**), and cyclic electron flow around photosystem I (ΔCEF) (**d**) on amplitudes of electrical signals in the leaflet of the second mature leaf (A_3_) in pea seedlings. Results of measurements in control pea seedlings, seedlings after 2 days of soil water shortage, and seedlings after 4 days of this shortage were analyzed together (*n* = 18). It was assumed that negative amplitudes corresponded to the hyperpolarization signal. R is the linear correlation coefficient.

Considering our earlier investigations of the influence of the burning-induced electrical signals on photosynthesis in pea seedlings [50,51,52,55], it was probable that ESs were a link between the burned zone and the photosynthetic responses in the non-damaged leaf of plant. Analysis of the correlations between the amplitudes of ESs in the second mature leaf (A_3_) and the magnitudes of changes in photosynthetic parameters (Figure 6) supported the influence of ESs on the photosynthetic parameters in the current variant of experiments. It was shown that these amplitudes were strongly correlated to ΔA_CO2_ (Figure 6a) and moderately correlated to ΔNPQ (Figure 6b) and ΔCEF (Figure 6d); all investigated correlation coefficients (excluding the weak correlation coefficient between A_3_ and ΔLEF, Figure 6c) were significant. The maximal absolute value of the correlation coefficient between A_3_ and ΔA_CO2_ was in a good accordance with our hypothesis about participation of inactivation of photosynthetic dark reactions in the induction of the photosynthetic response caused by ESs [7,44]. 

Figure 6 additionally supports that the hyperpolarization signals and depolarization signals with small amplitudes (<15 mV) were related to the stimulation of the photosynthetic CO_2_ assimilation; in contrast, depolarization signals with moderate and large amplitudes were related to the inactivation of this assimilation. It was probable that the changes in direction of ESs under water shortage (changes from VP to SP) could be the reason for the changes in direction of the response of A_CO2_ (changes from decrease of A_CO2_ to increase) after local burning under this shortage.

It should be noted additionally that the linear correlation coefficients between ΔA_CO2_ and ΔNPQ, ΔA_CO2_ and ΔLEF, and ΔA_CO2_ and ΔCEF, calculated on the basis of all measurements, were −0.81, 0.70, and 0.69, respectively (Figure S1); all coefficients were significant. Similar significant correlation coefficients were between ΔNPQ and ΔLEF (R = −0.91) and ΔNPQ and ΔCEF (R = 0.88) (Figure S2). 

### 2.4. Analysis of Participation of Changes in the Leaf Stomatal Conductance in Changes of the Photosynthetic CO_2_ Assimilation

Finally, we analyzed the participation of changes in g_H2O_ in the induction of changes in A_CO2_. The first question was: Why were there different directions of burning-induced changes in the leaf stomatal conductance? Earlier, we showed that the direction of ES-induced changes in the transpiration were related to the relative air humidity [33]. In the current experiment, this humidity was constant (about 70%); however, it was possible that variability in the initial g_H2O_ could be the additional factor influencing the direction of the response of the leaf stomatal conductance. 

**Figure 7:**
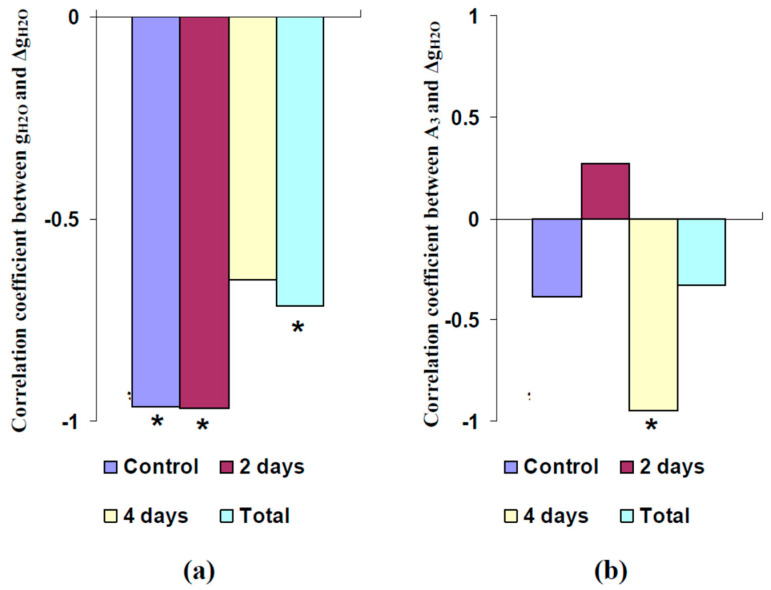
Correlation coefficients between the leaf stomatal conductance (g_H2O_) and its burning-induced changes (Δg_H2O_) (**a**) and between the amplitudes of the burning-induced electrical signals in the second mature leaf (A_3_) and Δg_H2O_ (**b**) in control pea seedlings (*n* = 6), seedlings after 2 days of soil water shortage (*n* = 6), seedlings after 4 days of this shortage (*n* = 6), and all investigated seedlings (*n* = 18). It was assumed that negative amplitudes corresponded to the hyperpolarization signal. *, the correlation coefficient was significant.

It was shown (Figure 7a) that initial g_H2O_ was significantly correlated with Δg_H2O_ in control pea seedlings, in seedlings after 2 days of soil water shortage, and in all investigated seedlings. However, this correlation was moderate and non-significant in seedlings after 4 days of water shortage. 

Analysis of correlations between Δg_H2O_ and A_3_ showed the opposite result (Figure 7b): a large and significant correlation between these parameters was only observed in seedlings after 4 days of soil water shortage (R = −0.95). These results showed that the variability of the initial values of g_H2O_ could be the main factor influencing the direction of the burning-induced changes under control conditions and moderate water shortage; in contrast, strong water shortage contributed to an increase of g_H2O_ after propagation of the electrical signals. 

**Figure 8:**
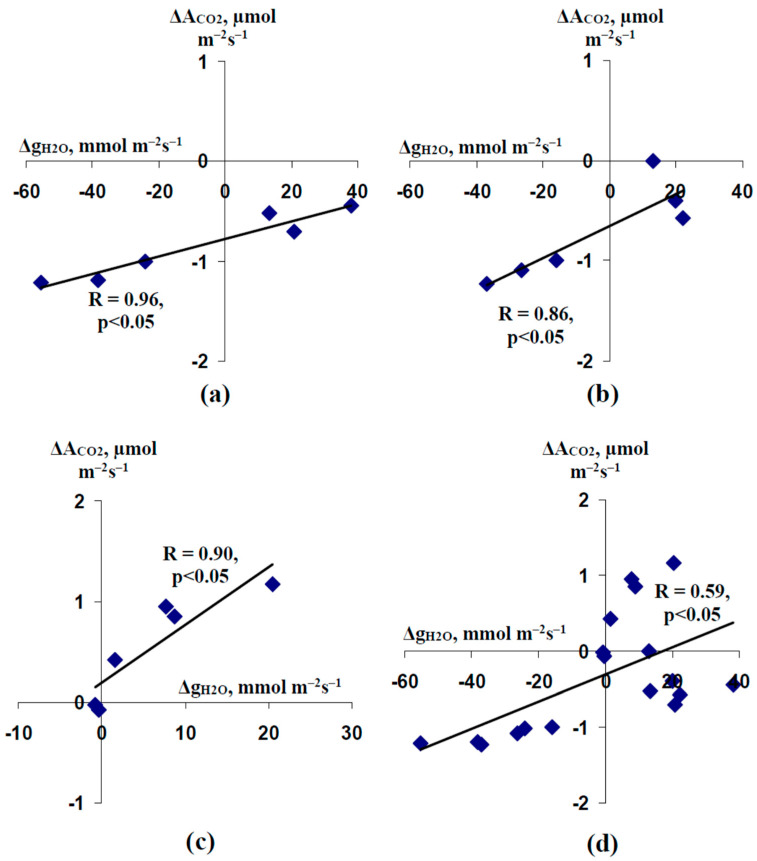
Dependences of burning-induced changes in the photosynthetic CO_2_ assimilation (ΔA_CO2_) on changes in the leaf stomatal conductance (Δg_H2O_) in control pea seedlings (*n* = 6) (**a**), seedlings after 2 days of soil water shortage (*n* = 6) (**b**), seedlings after 4 days of water shortage (*n* = 6) (**c**), and all investigated seedlings (*n* = 18) (**d**). R is the linear correlation coefficient.

Considering a strong relation between the amplitudes of ESs and the magnitudes of changes in g_H2O_ after 4 days of soil water shortage, we supposed that an increase of the leaf stomatal conductance could be the mechanism of activation of photosynthetic CO_2_ assimilation after 4 days of water shortage. Correlations between Δg_H2O_ and ΔA_CO2_ were investigated to check for this supposition (Figure 8).

It was shown that changes in the leaf stomatal conductance were positively and significantly related to changes in photosynthetic CO_2_ assimilation in all variants of analysis (Figure 8). However, these relationships did not influence ΔA_CO2_ in a qualitive manner under control conditions and after 2 days of soil water shortage, because stimulation of this assimilation was completely absent in these variants (Figure 8a,b). In contrast, increasing g_H2O_ was strongly related to increasing A_CO2_ in pea seedlings after 4 days of soil water shortage: ΔA_CO2_ was about zero at low Δg_H2O_ and ΔA_CO2_ was large and positive at large and positive Δg_H2O_ (Figure 8c). 

## 3. Discussion

ESs are an important mechanism of induction of the fast systemic physiological response under local actions of stressors [7,8]. The response can include fast changes in the expression of defense genes [19,20,21,22,23], production of stress phytohormones [23,24,25,26,27,28,29], water exchange [30,31,32,33,34], respiration [35,36,37], phloem transport [38,39,40,41,42], ATP content [43], and photosynthesis [44,45,46,47,48,49,50,51,52,53,54,55,56]. The result of these changes is an increase of the plant tolerance to the action of stressors [57,58,59,60,61,62,63,64,65,66]; it means that ESs can participate in the plant adaptation to changeable environmental factors. Modification of the parameters of ESs and ES-induced physiological changes under the long-term action of environmental stressors seems to be very probable because it can play an adaptive role; however, this problem has been weakly investigated. Earlier, we showed that plant electrical signals and ES-induced physiological changes can be modified by the long-term action of ionizing radiation [83]. The current work was devoted to analysis of the influence of soil water shortage, which was a model of soil drought (one of the key environmental factors for photosynthesis and productivity of plants [84,85,86,87]), on the burning-induced ESs and photosynthetic responses caused by these signals. 

**Figure 9:**
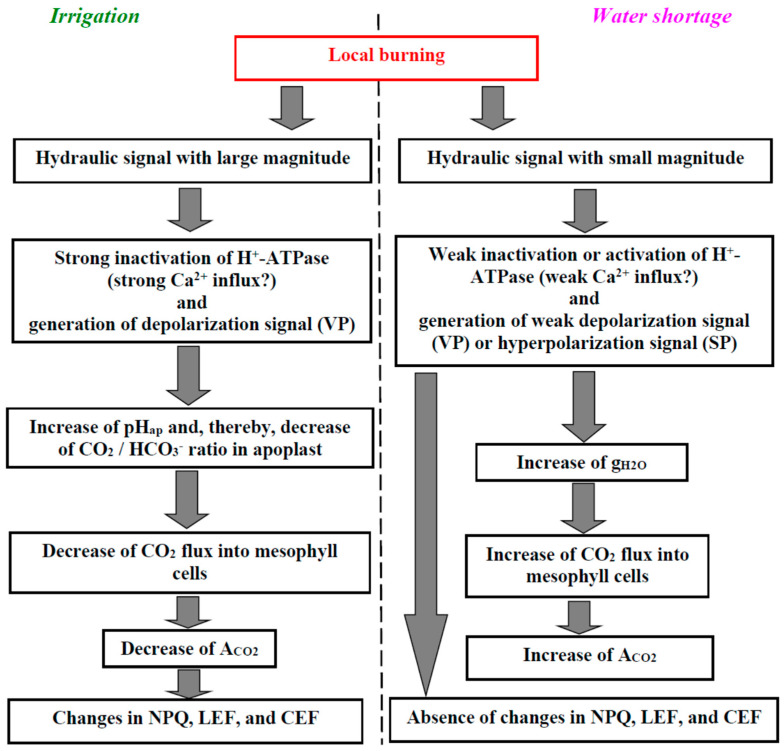
Hypothetical scheme of the influence of local burning on photosynthetic processes in non-damaged leaves under control conditions (irrigated plants) and under strong water shortage (see Section 3). See works [7,44] for details of ES influence on photosynthesis in irrigated plants; details of the ES influence on photosynthesis under water shortage are discussed in the text. pH_ap_ is the apoplastic pH.

There are several important points which are demonstrated in the current work (Figure 9). First, we showed that soil water shortage can strongly influence the parameters of burning-induced ESs including induction of inversion of direction of these electrical signals (Figure 2). There are several works [17,18,32,67,68,69] which show induction and propagation of hyperpolarization signals through the plant body. In accordance with Zimmermann et al. [17,18], these signals are system potentials which are related to the transient activation of H^+^-ATPase in the plasma membrane. It is important that these hyperpolarization signals can be observed under the actions of damages inducing typical depolarization signals. For example, local burning is known as an effective inductor of VP (the depolarization signal) in various plant species (see, e.g., [50,51,52,55,56] for pea, [47] for geranium, [100] for wheat, [54] for maize, [101] for mimosa, [26] for tobacco, [40] for *Vicia faba*, etc.); however, some works show that local burning can induce a hyperpolarization signal in maize [32,68] or poplar [69]. Moreover, the type of ES can be dependent on the localization of the burning [69]: a depolarization signal is observed after burning of the fourth leaf and a hyperpolarization signal is observed after burning of the first leaf. Our current results show that (i) the probability of propagation of the hyperpolarization signal can be stimulated by strong water shortage (however, depolarization signals can also be observed in some plants in this case) and (ii) the depolarization signal in the plant stem can be transformed into a hyperpolarization signal in the plant leaf. These results show that the type of ESs induced by the local damage (burning) in plants (the hyperpolarization or depolarization signals) is dependent on intricate mechanisms. 

Propagation of the wave of increased water pressure through xylem (a hydraulic signal), which transiently inactivates H^+^-ATPase in the plasma membrane (probably, by means of activation of mechanosensitive Ca^2+^ channels and influx of calcium ions into the cytoplasm), is mainly considered to be the potential mechanism of propagation of burning- or heating-induced variation potentials [1,9,10,102,103,104,105]. It is known [106,107] that water shortage (drought) decreases hydraulic pressure in plants; this decrease of initial pressure can decrease the value of the maximum hydraulic pressure after local burning. Additionally, the decrease of water content in plants under water shortage can decrease the magnitude of the burning-induced changes in the hydraulic pressure because the burning-induced water flux from the cells to xylem should be decreased (this flux is caused by efflux of osmolytes from damaged cells [105] and is dependent on the water content in the nearest cells). Considering the relation between the magnitude of the pressure increase and the amplitude of VP [102], this decrease of the pressure maximal value and magnitude of the pressure change should decrease the amplitude of VP under soil water shortage probably through a decrease of the magnitude of inactivation of H^+^-ATPase, which is the main mechanism of VP [1,2,7,8,9,10]. 

This mechanism explains a part of our results (decreased amplitude of VP under water shortage, Figure 2 and Figure 3); however, the inversion of ES direction in leaf under strong water shortage requires an additional explanation. Activation of H^+^-ATPase at low magnitudes of the hydraulic signal and its inactivation at moderate and high magnitudes seem to be the mechanism which can explain the induction of both the depolarization and hyperpolarization signals. There are some arguments supporting this hypothesis. (i) It has been shown that the increased pressure can activate H^+^-ATPase in the root cells of trees [108]. (ii) Ca^2+^ influx is the probable mechanism of influence of the hydraulic signal on the activity of H^+^-ATPase [10]. (iii) The increased Ca^2+^ concentration suppresses the activity of H^+^-ATPase [109]; however, there are works [110,111] showing that Ca^2+^ can induce the transition from inactive to active state of H^+^-ATPase (at least, under salt stress). (iv) The hypothesis about the two-phase dependence of activity of H^+^-ATPase on Ca^2+^ concentration (activation under moderate concentrations and inactivation under high concentrations) effectively explains the influence of a low-frequency magnetic field on the plant electrical activity [112].

Second, we show that water shortage can strongly influence ES-induced changes in the photosynthetic CO_2_ assimilation (Figure 4 and Figure 5). This influence is probably based on water shortage-induced changes in the amplitude of ESs (Figure 6). It is important that the inversion of direction of ESs or a strong decrease of their amplitude is accompanied by the inversion of the direction of changes in A_CO2_ (activation of the photosynthetic CO_2_ assimilation is observed at hyperpolarization signals or depolarization signals with small amplitudes). This result supports the idea about the direct relation between the direction of ESs and photosynthetic changes, which is based on several works [32,67,68]; additionally, it is in a good accordance with our results about linear correlations between amplitudes of ESs and the magnitudes of photosynthetic changes [44]. In contrast, this result contradicts the data of work [69] which shows a decrease of photosynthetic CO_2_ assimilation after both the depolarization and hyperpolarization signals. Our results show that the increase of the leaf stomatal conductance is the potential mechanism of this activation of A_CO2_ in seedlings after 4 days of soil water shortage (Figure 8c). However, Δg_H2O_ does not crucially influence photosynthetic CO_2_ assimilation in the control seedlings (Figure 8a) and seedlings after 2 days of water shortage (Figure 8b); moreover, changes in g_H2O_ are weakly related to the amplitude of ESs in these cases (control and 2 days of water shortage) (Figure 7). The result can be explained by the low initial value of g_H2O_ after 4 days of soil water shortage—transport of CO_2_ through stomata is not the main limiting factor for photosynthesis under watered conditions or under weak water shortage [113,114]; however, it can be the limiting factor under strong water shortage and strong stomata closing. It means that the activation of A_CO2_ after propagation of the hyperpolarization signals can be absent under other conditions (without water shortage), and other responses (e.g., inactivation of A_CO2_ [69]) can be observed. 

Third, our results show that the magnitudes of ES-induced changes in the parameters of the photosynthetic light reactions (NPQ, LEF, and CEF) are strongly decreased with the development of water shortage (Figure 4 and Figure 5). This result can be explained by both the decrease of the amplitudes of the depolarization signals under water shortage (amplitudes of ESs are significantly correlated to ΔNPQ and ΔCEF, Figure 6) and the decrease of the initial values of the parameters (at least, the linear correlation coefficient between LEF and ΔLEF is significant and equals –0.63, data not shown). It should be noted that the correlations between ΔA_CO2_ and the parameters of photosynthetic light reactions (Figure S1) are stronger than the correlations of these parameters with the amplitudes of ESs; correlations between the parameters of photosynthetic light reactions (Figure S2) are stronger than the correlations of these parameters with ΔA_CO2_. This result shows that ESs primarily influence photosynthetic CO_2_ assimilation; after that, changes in this assimilation influence the photosynthetic light reactions. This chain of events is in good accordance with one of the ways of ES influence on photosynthesis, which has been shown in plants under irrigated conditions [7,43,44,46,47,51,52]: local damage, the generation and propagation of VP (the decrease of the H^+^-ATPase activity), the alkalization of the apoplast, the decrease of CO_2_/HCO_3_^-^ ratio, the decrease of the CO_2_ flux into mesophyll cells and suppression of photosynthetic dark reactions, the increase of ratios of ATP/ADP and NADPH/NADP^+^, the suppression of activity of H^+^-ATP-synthase in the thylakoid membrane, the increase of the luminal concentration of protons in the thylakoids of chloroplasts, the suppression of LEF, and stimulation of CEF and NPQ. It means that the suppression of ES-induced changes in NPQ, LEF, and CEF under soil water shortage can be mainly caused by the decrease of the magnitude of the A_CO2_ suppression in this case—participation of the direct influence of ESs on the photosynthetic light reactions in these water shortage-induced modifications is not probable.

It is considered [6,7,8,44,57,58,59,60] that ESs increase plant tolerance to environmental stressors. Particularly, ES induced adaptive changes in photosynthetic processes [6,7,8,44] including an increase of NPQ and CEF and a decrease of LEF and A_CO2_; the last response contributes to an increase of the ATP content in leaf and can be important for reparation of the photosynthetic machinery [43,91]. It can be expected that these changes are not crucial under stress conditions (soil water shortage in our work) because these protective mechanisms are activated earlier by a direct action of stressors (increased NPQ and decreased LEF and A_CO2_ are observed under water shortage conditions, Figure 1). Thus, our results show that a direct action of stressors (soil water shortage) can suppress ES-induced photosynthetic response; the result is in a good accordance with our previous work [83]. Additionally, the following is not clear: Can the activation of photosynthetic CO_2_ assimilation under strong water shortage, participate in the plant protection to stressors? We cannot fully exclude that the ES-induced activation of CO_2_ assimilation during the suppression of this process by strong water shortage can participate in an increase of plant tolerance to stressors (e.g., through additional synthesis of organic compounds including, maybe, osmotically active agents). However, this problem requires further investigation. 

## 4. Materials and Methods

### 4.1. Pea Cultivation and Water Shortage Induction

Seedlings of 2–3-week-old pea (*Pisum sativum* L., cultivar “Albumen”, Central Experimental and Production Facility of Roika, Roika, Russia) were investigated. The seedlings were cultivated in 400 mL pots with a sand substrate (about 350 g) in a growth room under 16/8 h (light/dark) photoperiod at 24 °C. There were six pea seedlings per pot. Plants were irrigated by 50% Hoagland–Arnon medium every 2 days (about 50 mL). 

Termination of the irrigation of experimental seedlings was used for fast induction of soil water shortage; control seedlings were irrigated. In accordance with our previous results, which were shown in similar conditions [88,89,115], this termination induced a decrease of the relative water content in the sand substrate (calculated as the ratio of the difference between fresh and dry weights of sand to its fresh weight) from 10–12% (irrigated pots) to less than 0.5% after 2 and 4 days of water shortage. It was previously shown [89,115] that this water shortage decreased the relative water content in leaves by about 2% after 2 days of water shortage and by about 10% after 4 days of water shortage. Visual estimation showed that seedlings had decreased turgor in the leaves after 4 days of water shortage. 

Electrical signals, photosynthetic processes, and leaf stomatal conductance were investigated in seedlings after 2 and 4 days after termination of irrigation. It should be noted that control seedlings were irrigated on the days above before the measurements. 

### 4.2. Local Burning and Measurements of Electrical Signals

**Figure 10:**
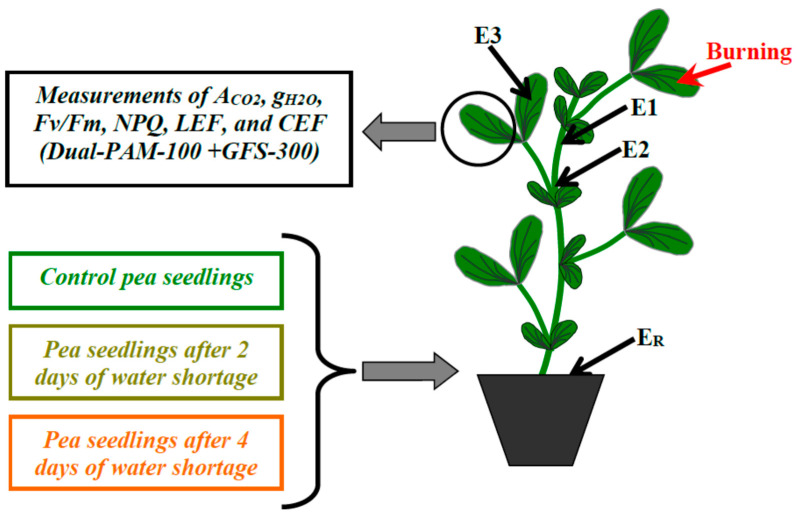
Scheme of measurements of surface electrical potentials, photosynthetic parameters, and leaf stomatal conductance in pea seedlings under control conditions and after 2 and 4 days of soil water shortage. A_CO2_ is the photosynthetic CO_2_ assimilation; g_H2O_ is the leaf stomatal conductance; Fv/Fm is the maximal quantum yield of photosystem II; NPQ is the non-photochemical quenching; LEF is the photosynthetic linear electron flow; and CEF is the cyclic electron flow around photosystem I. E1, E2, and E3 are the measuring electrodes; E_R_ is the reference electrode. The water shortage was initiated by termination of irrigation of the plants. The red arrow marks the local burning of the first mature leaf (flame, 2–3 s).

The local burning of the first mature leaf (open flame, 3–4 s, about 1 cm^2^) was used for induction of ESs (Figure 10) in accordance with our previous works (e.g., [34,43,52,55,56]). This burning was induced after a 1.5 h adaptation of the plants in the experimental set because this adaptation duration (about 1.5 h) was considered to be enough for the induction of electrical signals and photosynthetic responses in pea seedlings [43,50,51,52,55,56]. 

ESs were measured using extracellular Ag^+^/AgCl electrodes (RUE Gomel Measuring Equipment Plant, Gomel, Belarus), a high-impedance IPL-113 amplifier (Semico, Novosibirsk, Russia), and a personal computer. The measuring electrodes were contacted to the stem near to the first mature leaf (E1), the stem near to the second mature leaf (E2), and the leaflet of the second mature leaf (E3). The electrodes were contacted to a plant by cotton threads wetted with a standard solution (1 mM KCl, 0,5 mM CaCl_2_, 0,1 mM NaCl) in accordance with our previous works [29,83]. The reference electrode (E_R_) was contacted to the growth substrate. 

### 4.3. Measurements of Photosynthetic Parameters and Leaf Stomatal Conductance 

A GFS-3000 gas analyzer (Heinz Walz GmbH, Effeltrich, Germany), Dual-PAM-100 Pulse-Amplitude-Modulation (PAM)-fluorometer (Heinz Walz GmbH, Effeltrich, Germany), and Dual-PAM gas-exchange Cuvette 3010-Dual common measuring head (Heinz Walz GmbH, Effeltrich, Germany) were used for investigations of photosynthetic parameters and leaf stomatal conductance (Figure 10). Photosynthetic measurements were performed on the second mature leaf. 

The concentration of CO_2_ in the measuring cuvette, relative humidity, and temperature were 360 ppm, 70%, and 23 °C, respectively. Blue actinic light (460 nm, 240 µmol m^−2^s^−1^) was used in the investigation. Photosynthetic measurements were initiated after 20 min dark intervals; the initial and maximum levels of photosystem II fluorescence (F_0_ and F_m_, respectively) and maximum light absorption by photosystem I (P_m_) were measured after dark adaptation. The current levels of fluorescence (F), maximum fluorescence level after the preliminary illumination (F_m_’), current light absorption by photosystem I (P), and maximum light absorption by photosystem I after the preliminary illumination (P_m_’) were measured for each saturation pulse; saturation pulses were generated every 30 s. These parameters were used for the calculation of NPQ and the quantum yields of photosystem I (Φ_PSI_) and photosystem II (Φ_PSII_) in accordance with the standard equations [116,117,118]. 

LEF and CEF were calculated based on Equations (1) and (2) [50,51,53]:

(1) $$\mathrm{LEF}=\beta \times \mathrm{PAR}\times \mathrm{dII}\times \Phi_{\mathrm{PSII}}$$

(2) $$\mathrm{CEF}=\beta \times \mathrm{PAR}\times \left[\left(1-\mathrm{dII}\right)\times \Phi_{\mathrm{PSI}}-\mathrm{dII}\times \Phi_{\mathrm{PSII}}\right]$$

where PAR is the intensity of the actinic light, β is the fraction of the actinic light absorbed by the leaves equal to 0.88 in accordance with [51], dII is the fraction of the absorbed light distributed to photosystem II, and (1–dII) is the fraction of the absorbed light distributed to photosystem I. In accordance with the earlier proposed method [50,51], dII was calculated as $\frac{\Phi_{\mathrm{PSI}}}{\Phi_{\mathrm{PSI}}+\Phi_{\mathrm{PSII}}}$, where both Φ_PSI_ and Φ_PSII_ were measured under a low intensity of actinic light. 

GFS-3000 (Heinz Walz GmbH, Effeltrich, Germany) was used for the measurements of CO_2_ assimilation and leaf stomatal conductance which were automatically calculated by GFS-3000 software. A_CO2_ was calculated as the difference between CO_2_ assimilation (A) under light and dark conditions.

The actinic light was initiated at 2 min after the start of the generation of the saturation pulses. The local burning was induced after 108 min of illumination; photosynthetic parameters without ESs were measured before the local burning (after about 107 min of illumination). ΔA_CO2_, ΔNPQ, ΔLEF, and ΔCEF were calculated as the difference between the extremes of these parameters and their values before the induction of the ESs.

### 4.4. Statistics 

Different seedlings were used for each experiment; *n* was their quantity which equaled 6 for each experimental variant and 18 for the correlation analysis on the basis of all the experimental variants. Averaged values, standard errors, representative records, scatter plots, and linear correlation coefficients (Pearson correlation coefficients) were presented. The linear correlation coefficients (and linear regressions describing the scatter plots) were used because they were the simplest criteria of the relations between the investigated parameters and were suitable for comparison between different relations. The significance of differences was estimated using the Student’s *t*-test (for *p* < 0.05). Microsoft Excel 365 was used for statistical analysis. 

## 5. Conclusions

The current work was devoted to analysis of the influence of soil water shortage on the burning-induced electrical signals and ESs-induced changes in photosynthetic parameters. Three important points were illustrated. First, development of soil water shortage decreased the amplitudes of the burning-induced ESs and, even, contributed to the propagation of the hyperpolarization signals under strong water shortage. Second, development of soil water shortage decreased the ES-induced response of photosynthetic CO_2_ assimilation which was strongly related to the amplitudes of the electrical signals in investigated leaves. The direction of this response was changed under strong water shortage (inactivation of CO_2_ assimilation was observed in control seedlings and seedlings after 2 days of water shortage and activation of this assimilation was observed in seedlings after 4 days of water shortage). Activation of photosynthetic CO_2_ assimilation was probably caused by the ES-induced increase of leaf stomatal conductance under strong water shortage. Third, the soil water shortage development decreased the magnitudes of changes in the parameters of the photosynthetic light reactions (the non-photochemical quenching, linear electron flow and cyclic electron flow around photosystem I) induced by the local burning and propagation of ESs. 

Thus, our results show that long-term action of environmental stressors (soil water shortage) can modify the damage-induced electrical signals and photosynthetic responses caused by these signals. These modifications can be an additional mechanism adaptation for higher plants to the changeable environmental conditions. 

## Data Availability

The data presented in this study are available on request from the corresponding author.

## Supplementary Materials

The following supporting information can be downloaded at: https://www.mdpi.com/article/10.3390/plants11040534/s1, Figure S1: Dependences of burning-induced changes in non-photochemical quenching (ΔNPQ) (**a**), photosynthetic linear electron flow (ΔLEF) (**b**), and cyclic electron flow around photosystem I (ΔCEF) (**c**) on burning-induced changes in the photosynthetic CO_2_ assimilation (ΔA_CO2_); Figure S2: Dependences of burning-induced changes in the photosynthetic linear electron flow (ΔLEF) (**a**), and cyclic electron flow around photosystem I (ΔCEF) (**b**) on burning-induced changes in non-photochemical quenching (ΔNPQ).
