# Supplementary material for: Influence of Burning-Induced Electrical Signals on Photosynthesis in Pea Can Be Modified by Soil Water Shortage
Source: Plants (Basel). 2022 Feb 17;11(4):534. doi: 10.3390/plants11040534 (PMC8878130; doi:10.3390/plants11040534)
Supplement: Supplementary file 1 [file plants-11-00534-s001.zip › Figure S1.pdf]

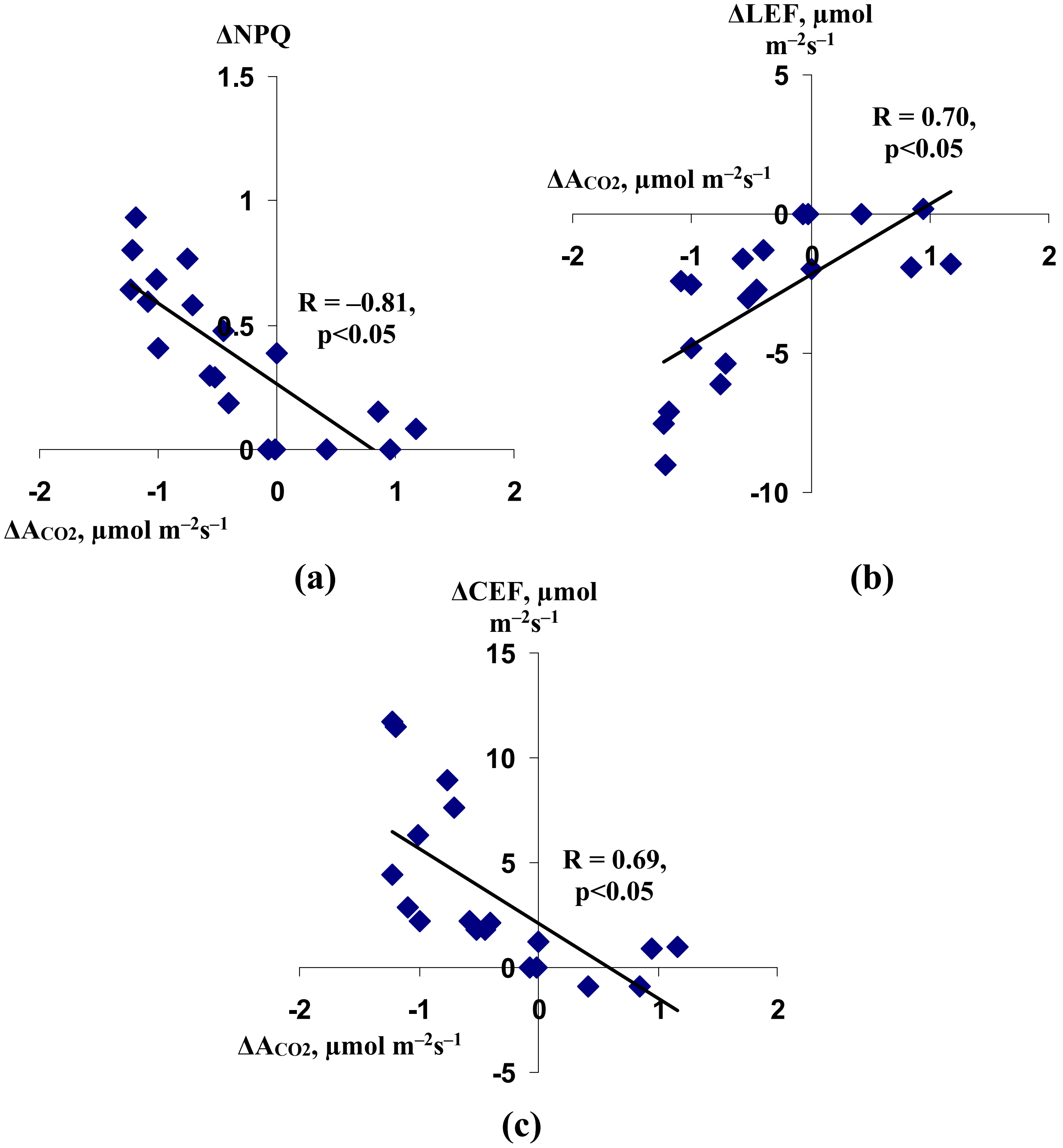

Figure S1. Dependences of burning-induced changes in the non-photochemical quenching (ΔNPQ) **(a)**, photosynthetic linear electron flow (ΔLEF) **(b)**, and cyclic electron flow around photosystem I (ΔCEF) **(c)** on burning-induced changes in the photosynthetic CO_2 assimilation (ΔA_{CO2}). Results of measurements in control pea seedlings, seedlings after 2 days of the water shortage, and seedlings after 4 days of this shortage were together analyzed (*n*=18). R is the linear correlation coefficient.
