# Supplementary material for: Influence of Burning-Induced Electrical Signals on Photosynthesis in Pea Can Be Modified by Soil Water Shortage
Source: Plants (Basel). 2022 Feb 17;11(4):534. doi: 10.3390/plants11040534 (PMC8878130; doi:10.3390/plants11040534)
Supplement: Supplementary file 1 [file plants-11-00534-s001.zip › Figure S2.pdf]

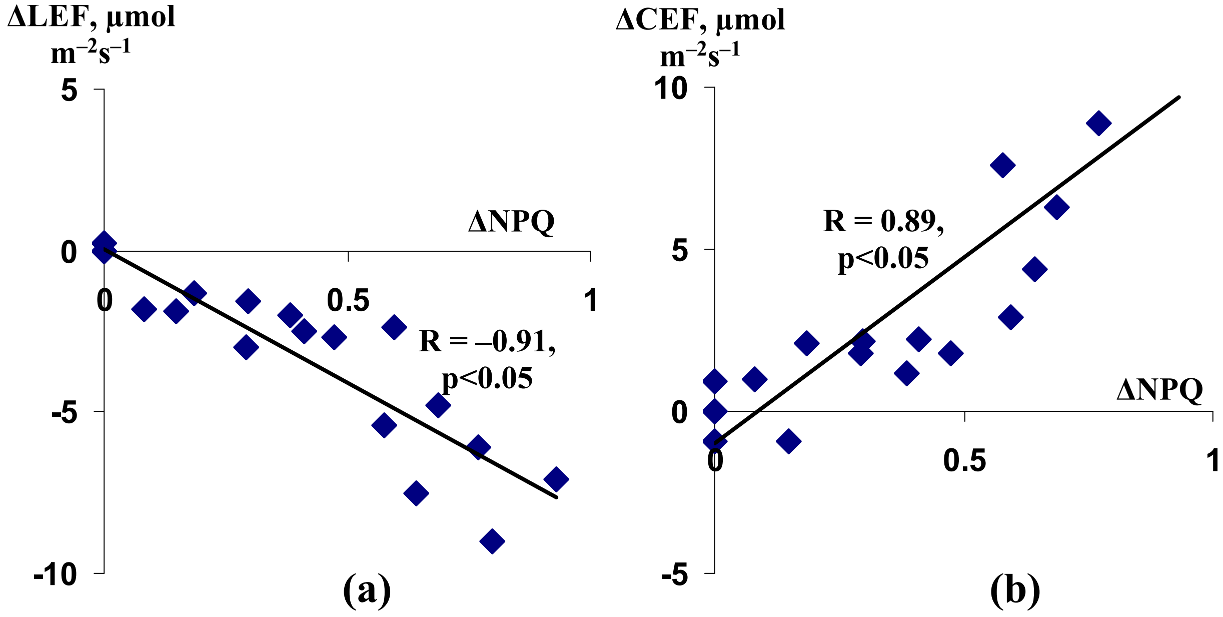

Figure S2. Dependences of burning-induced changes in the photosynthetic linear electron flow (ΔLEF) **(a)**, and cyclic electron flow around photosystem I (ΔCEF) **(b)** on burning-induced changes in non-photochemical quenching (ΔNPQ). Results of measurements in control pea seedlings, seedlings after 2 days of the water shortage, and seedlings after 4 days of this shortage were together analyzed (n=18). R is the linear correlation coefficient.
